# Sensitivity analysis of the precooling process of strawberry: Effect of package designing parameters and the moisture loss

**DOI:** 10.1002/fsn3.1536

**Published:** 2020-03-24

**Authors:** Habibeh Nalbandi, Sadegh Seiiedlou

**Affiliations:** ^1^ Department of Biosystem Engineering Faculty of Agriculture University of Tabriz Tabriz Iran

**Keywords:** heterogeneity, package design, parallel airflow system, precooling, simulation, strawberry

## Abstract

Strawberry is one of the most perishable fruits, and precooling of strawberry increases the percentage of marketable fruits. To assess the sensitivity of strawberry cooling uniformity with respect to package vents and tray design, the previously proposed modified parallel airflow system (MPAS) was modified and a sensitivity analysis was conducted in this paper. Some improvements in homogeneous strawberry precooling process were made to give improved parallel airflow system (IPAS). To evaluate its performance, the cooling process of strawberries was simulated using the mathematical models of heat, momentum, and mass transfer, which were validated experimentally. Results showed that the IPAS was able to distribute cold air uniformly throughout the packages. A difference of 0.1°C was observed between the average fruit temperatures of the packages after 3 hr of cooling. Therefore, the cooling process of strawberry could be done at lower airflow rate, cooling time, and heterogeneity. In addition, the precooling process of strawberry was studied considering the moisture loss during the cooling process and comparing the data with the models without this term. The results indicated that the moisture loss of strawberries during the cooling process is not negligible and the cooling rate increased and the cooling time decreased (31%) by considering this term in the modeling. However, the moisture loss did not affect the heterogeneity of cooling process.

## Graphical abstract

Improved parallel airflow system was able to distribute cold air uniformly throughout the packages. The cooling process of strawberry could be done at lower airflow rate, cooling time, and heterogeneity compared with previous systems.

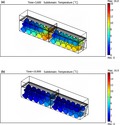

## 1 INTRODUCTION

Temperature management is a key factor in maintaining fruits and vegetable quality and extending their shelf life. Delays in cooling for more than 2 hr reduce both the fruit quality during the storage and the percentage of marketable fruits. Thus, it is essential to remove rapidly field heat from freshly harvested fruit prior to storage. During the precooling process, the field temperature of the fruit is lowered to the ^7^/_8_th storage temperature immediately after harvest (Anderson, Sarkar, Thompson, & Singh, 2004; Brosnan & Sun, 2001; Chakraverty & Paul, 2001; Kader, 2002; Kumar, Kumar, & Murthy, 2008; Manganaris, Iliasb, Vasilakakisa, & Mignanic, 2007). Poor airflow distribution among different locations in the package leads to considerable heterogeneities in the final temperature of the product (Alvarez & Flick, 1999a, 1999b; Amara, Laguerre, & Flick, 2004). Many researchers have studied the effect of package design and pallet arrangements on the cooling rate and heterogeneity of cooling process. Wu, Haller, Cronje, and Defraeye (2018) investigated the cooling rate and heterogeneity of packed citrus fruit in a full‐scale, forced‐air precooling system and the effects of package design and fruit size was studied on the precooling performance. They reported that the cooling heterogeneity occurred mainly along the flow direction. Fruit wrapping induced a much slower cooling rate and larger cooling heterogeneity, especially in cartons at the outflow side of the pallet. The cooling process of orange and tomato, kiwifruit, and pomegranate fruit were also studied by Kumar et al. (2008), O'Sullivan et al. (2016a, 2016b), and Mukama, Ambaw, and Opara (2019), respectively.

**FIGURE 1:**
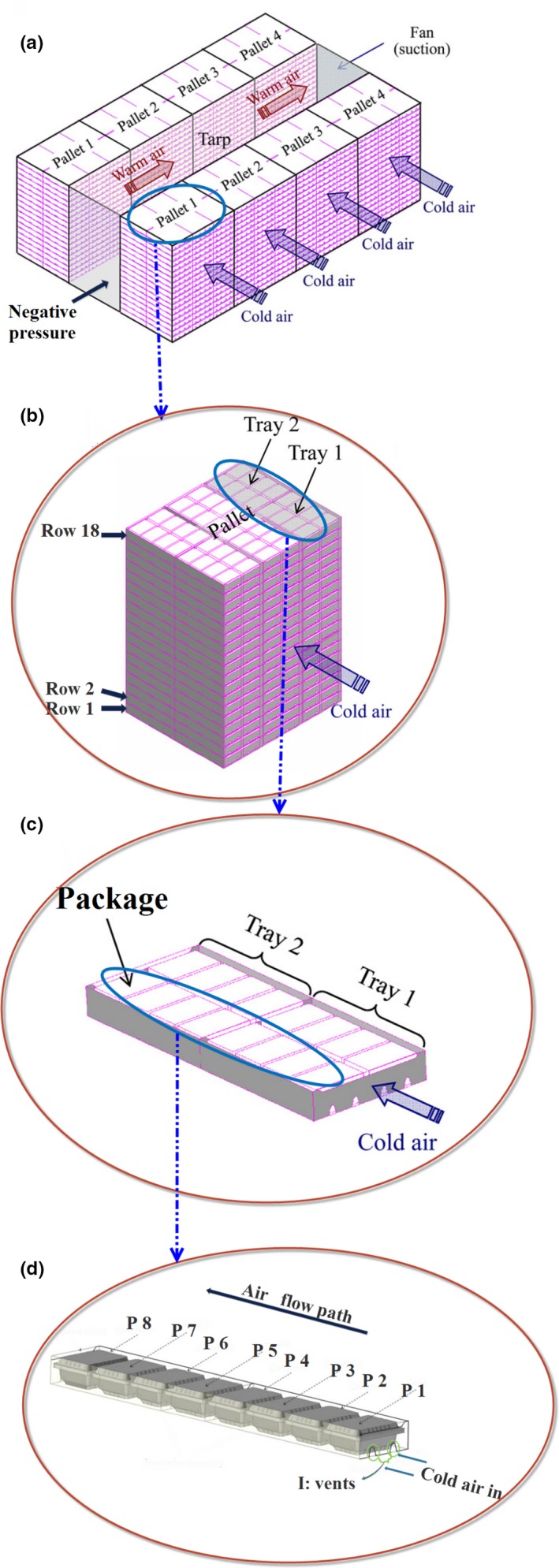
The industrial forced‐air cooling system of strawberries (Ferrua & Singh, 2009d)

Among the fruits, strawberry is one of the most appreciated fruits in the world and is popular for its pleasant flavors and nutritional qualities (Liu et al., 2016). However, it is one of the most perishable fruits and is susceptible to mechanical damage and water loss. Precooling of strawberry maintains its quality by reducing its respiration and transpiration rates (Anderson et al., 2004). This process was carried out by the forced‐air cooling system shown in Figure 1a; the cold air is forced across each palletized row (Figure 1b,c) through the vents in the trays and packages (I in Figure 1d) due to the negative relative pressure created by the system suction fan (Ferrua & Singh, 2009d).

Ferrua and Singh (2009a, 2009b, 2009c, 2009d) reported that in the industrial precooling of strawberries (Figure 1), there was up to about 6°C difference in average fruit temperatures between the packages after 1 hr of cooling (airflow rate: 1 L s^−1^ kg_p_^−1^), and about 75% of total airflow bypassed the packages. To decrease the cooling heterogeneity, they developed a novel forced‐air cooling system that split the airflow in each layer of the pallet into two streams of parallel airflows (Ferrua & Singh, 2011). This system somehow improved the uniformity of cooling between two trays and reduced the cooling time (by 6%), pressure drop, and energy consumption. Despite their relative success, there is still a considerable heterogeneity in the cooling process of strawberries.

**FIGURE 2:**
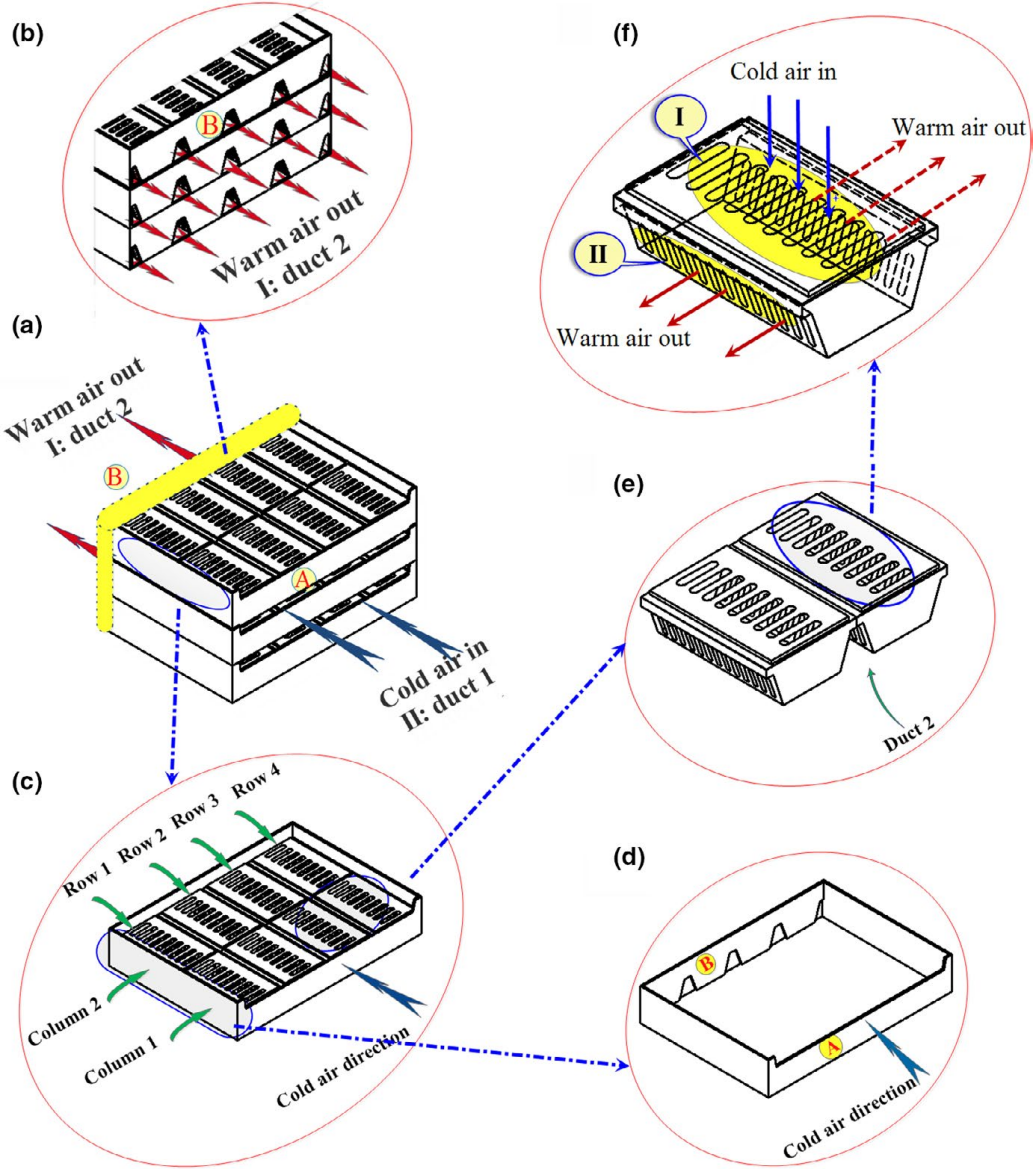
Parallel airflow system (PAS) for forced‐air cooling of strawberries; duct 1: entrance cold air; ducts 2: exhaust warm air; A: entrance side from duct 1; B: exhaust side to duct 2; I: entrance vents to packages; II: exhaust vents from packages (Nalbandi et al., 2016)

Nalbandi, Seiiedlou, Ghassemzadeh, and Ranjbar (2016) introduced an innovative parallel airflow system (PAS) for forced‐air cooling of strawberries to improve the homogeneity of strawberries precooling process (Figure 2). In the proposed system, two separate ducts were designed for introducing the cold air into the individual fresh fruit packages in a way that the exiting heated air can return to air cooling unit without re‐entering other packages. One of these ducts (duct 1) was built on the top of the packages, and the other one (duct 2) was located between the packages (Figure 2a). The cold air enters duct 1 before entering the packages where it removes heat from the fruits and exits through ducts 2. To achieve this goal, they designed a new tray with related vents (Figure 2d). Duct 1 was created by constructing the tray wall taller than the package walls (Figure 2c), and duct 2 was created by considering a trapezoidal cross section for the strawberry package (Figure 2e). The related vents for ducts 1 and 2 were considered on the tray walls. The vent in side A is related to duct 1 on the top of the packages, and the trapezoidal vents in side B is referred to duct 2 between the packages (Figure 2a,b,d). The location and shape of the vents on package walls play an important role in PAS because of relating the inlet and outlet ducts. The vents were distributed on the top and lateral walls of packages as inlet (I in Figure 2f) and outlet vents (II in Figure 2f).

When the suction fan is turned on, the cold airstream first enters duct 1, then passes through top openings of packages (I in Figure 2f), and exits finally through openings on the walls of the packages (II in Figure 2f). Heated airstream returns to cold room for recirculation through duct 2. They simulated the airflow and heat transfer inside the developed system.

**FIGURE 3:**
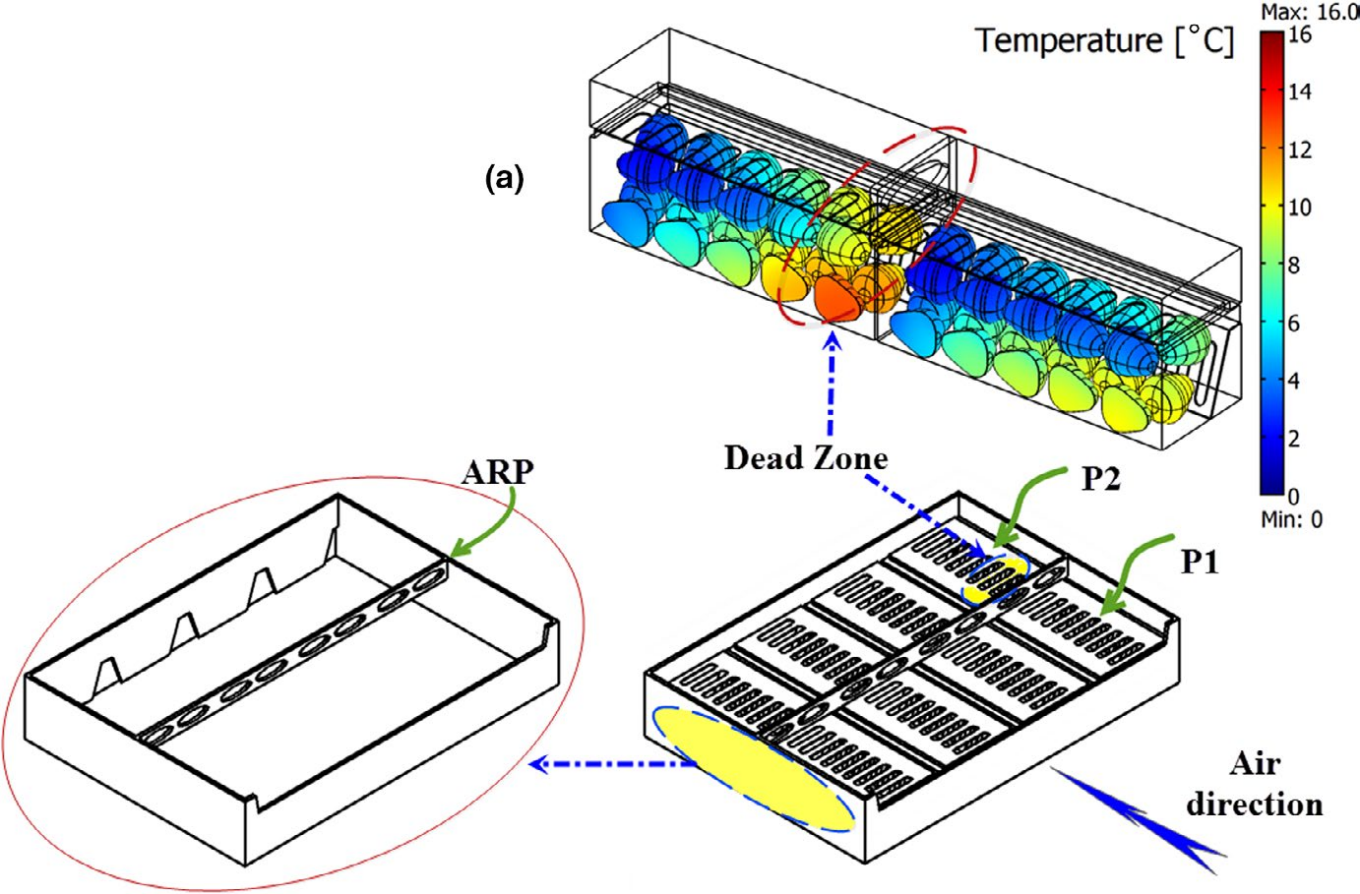
Modified parallel airflow system (MPAS); ARP is the airflow restriction plate; P1and P2 are packages 1 and 2, respectively; different colors in (a) shows different temperatures of fruits (Nalbandi et al., 2016)

According to the results, the parallel airflow system (PAS) was improved by inserting an airflow restriction plate “ARP,” that is, a plate with holes on it (Figure 3). They introduced their new system as modified parallel airflow system (MPAS). In MPAS, the cold air was divided uniformly between the packages and they received about 46 and 54% of total airflow rate. The cooling process of fruits inside the packages was performed more uniformly, and a 0.84°C temperature difference was observed between the average fruit temperature of the packages after 3 hr of cooling process. However, their results indicated that the inlet vents located behind the ARP (Figure 3) received a lower airflow rate (about 25% of total airflow rate entered package 2). This area was introduced as a dead zone, and fruits located at this zone were cooled more slowly (Figure 3); as a result, about 2.5°C temperature difference was observed between fruit temperatures. The shape of ARP could create such uniformity in temperature. ARP had two vents across each package creating a dead zone behind the middle plane of package 2 (P2). In addition, the results showed some differences between the model and experimental data. One justification for this error is related to the model assumption; the respiration process was assumed negligible, and there would be no shrinkage or moisture loss during the cooling process.

Mercier, Brecht, and Uysal (2019) investigated the product temperature distribution during the commercial forced‐air precooling of strawberries. They found that the half‐cooling time of fruit located near the central location of tunnels was approximately twice that of fruit located on the outside facing the incoming air. Temperature variations of up to 7°C were observed at the end of precooling, suggesting that precooling should be extended in some instances to improve uniformity.

A review of lectures showed that, in the traditional cooling system of strawberry, there is considerable heterogeneity between the fruits temperature located in the individual packages and in different packages inside the tray, which leads to a decrease in the self‐life of fruits. The efforts of researchers could not solve this problem properly. However, Nalbandi et al. (2016) showed that the package and tray design had a main role in improving the cooling uniformity. Therefore, the effects of different designs of tray and the extra vent designing on the package walls were studied on the strawberry cooling efficiency.

The aims of this research were (a) to assess the sensitivity of strawberry cooling uniformity with respect to package vent and tray design, (b) to simulate the effectiveness of the proposed process using a previously validated model by Nalbandi et al. (2016), and (c) to study the moisture loss of strawberry after harvest and modifying the developed mathematical model by considering the moisture loss term.

## 2 MATERIALS AND METHODS

### 2.1 Design of Airflow Restriction Plate

**FIGURE 4:**
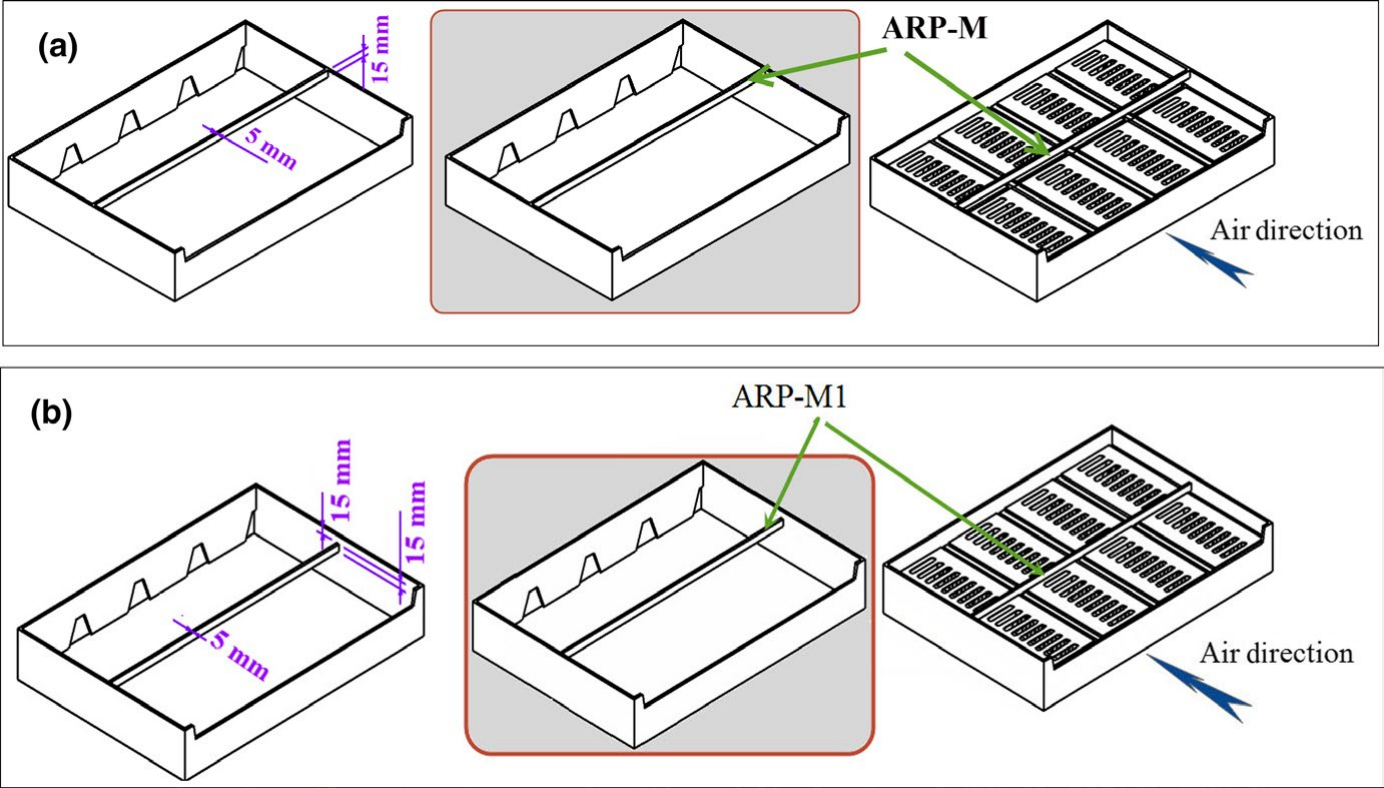
Various designs of the airflow restriction plate (ARP); (a): ARP‐M, (b): ARP‐M1

To eliminate the dead zone created between the packages in the previous study (Nalbandi et al., 2016), various designs for the airflow restriction plate (ARP) were studied with respect to its dimension and location. A primary study indicated that the velocity of cold air increased when it passed through the ARP vents. High velocity did not let air streams to change their direction. Therefore, the vents of ARP were emitted and ARP‐M was designed herein (Figure 4a). Width and thickness of ARP‐M were 5 and 15 mm, respectively, and its location is illustrated in Figure 4a. ARP‐M could not solve the problem and emitted the dead zone (results were not shown). At the next stage, the location of ARP‐M was changed and named ARP‐M1. It was located with 15 mm distance from the upper face of the tray close to the package lid (Figure 4b). The cooling process of strawberry using the new tray consisting of ARP‐M1 is described in the following section.

### 2.2 Simulation of the cooling process of strawberries using ARP‐M1

To evaluate the performance of ARP‐M1, the cooling process of strawberries was simulated based on the mathematical modeling of airflow and heat transfer between airstream and strawberries, which was validated experimentally by Nalbandi et al. (2016). The equations and related boundary conditions are summarized in Table 1. Equations 1 and 2 represent the mass and momentum conservation for air in the laminar flow regime. The flow rate was assumed 0.4 L s^−1^ kg_p_^−1^, and a Reynolds number of 54 was calculated inside the packages. Equation 3, consisting of conduction, and convection transient heat transfer, modeled heat transfer inside the fluid domain. Equation 4 describes the conduction heat transfer in the product domain. In the fruit‐air interface, Equation 5 was used as a boundary condition. Other boundary and initial conditions, as well as strawberry and air properties, are reported in Table 1.

**Table 1:** Mathematical models and related boundary conditions

Airflow model	Incompressible fluid and laminar flow	∇.u=0 (1) $\rho_{a}\frac{\partial u}{\partial t}+\rho_{a}\left(u.\nabla u\right)=-\nabla P+\nabla .\;\;\left[\;\mu_{a}\left(\nabla u+{\left(\nabla u\right)}^{T}\right)\right]$ (2)
	Reynolds number	Inside the packages: 54 At the inlet: 710
	Boundary conditions	Inlet: *p* = *p*_0_ Wall: **u** = 0 Outlet: **u** = **u**_0_ Interface: **u** = 0
	Airflow rate	0.4 L s^−1^ kg_p_^−1^
	Air properties	Similar to those of dry air at 0°C
	Transient heat transfer	$\rho_{a}Cp_{a}\frac{\partial T_{a}}{\partial t}+\rho_{a}Cp_{a}\left(u\;.\nabla T_{a}\right)=\nabla .\;\left(k_{a}\nabla T_{a}\right)$ (3)
Heat transfer within the fluid domain	Boundary conditions	Inlet: *T*_a_ = *T*_a0_ Outlet: $\left(-k_{a}\nabla T_{a}\right)n=0$ Wall: $\left(k_{a}\nabla T_{a}\right)\;\;n=0$ Interface: T_a_ = T_p_ Symmetry plane: $\left(k_{a}\nabla T_{a}\right)n=0$
	Air properties	Similar to those of dry air at 0°C
	Transient heat transfer	$\rho_{p}\;Cp_{p}\frac{\partial T_{p}}{\partial t}=\nabla .\;\;\left(k_{p}\nabla T_{p}\right)$ (4)
Heat transfer in the strawberries domain	Boundary conditions	$\left(k_{P}\nabla T_{P}-k_{a}\nabla T_{a}\right)\;\;n=0$ (5)
	Strawberry properties	*k*_p_ = 0.57 wm^−1^°C^−1^ *C*_p,_ *_p_* = 3.95 kJ/kg °C *ρ*_p_ = 800 kg/m^3^
	Initial temperature	16°C
	Assumption	No moisture loss during the cooling

Note

In the above equations, *C_p_* and *ρ* denote the specific heat capacity and density, respectively **u** and *P* represent the air velocity and pressure, respectively; *k* is thermal conductivity; and *T* shows the temperature. Subscripts *a* and *p* indicate the air and the product, respectively.

**FIGURE 5:**
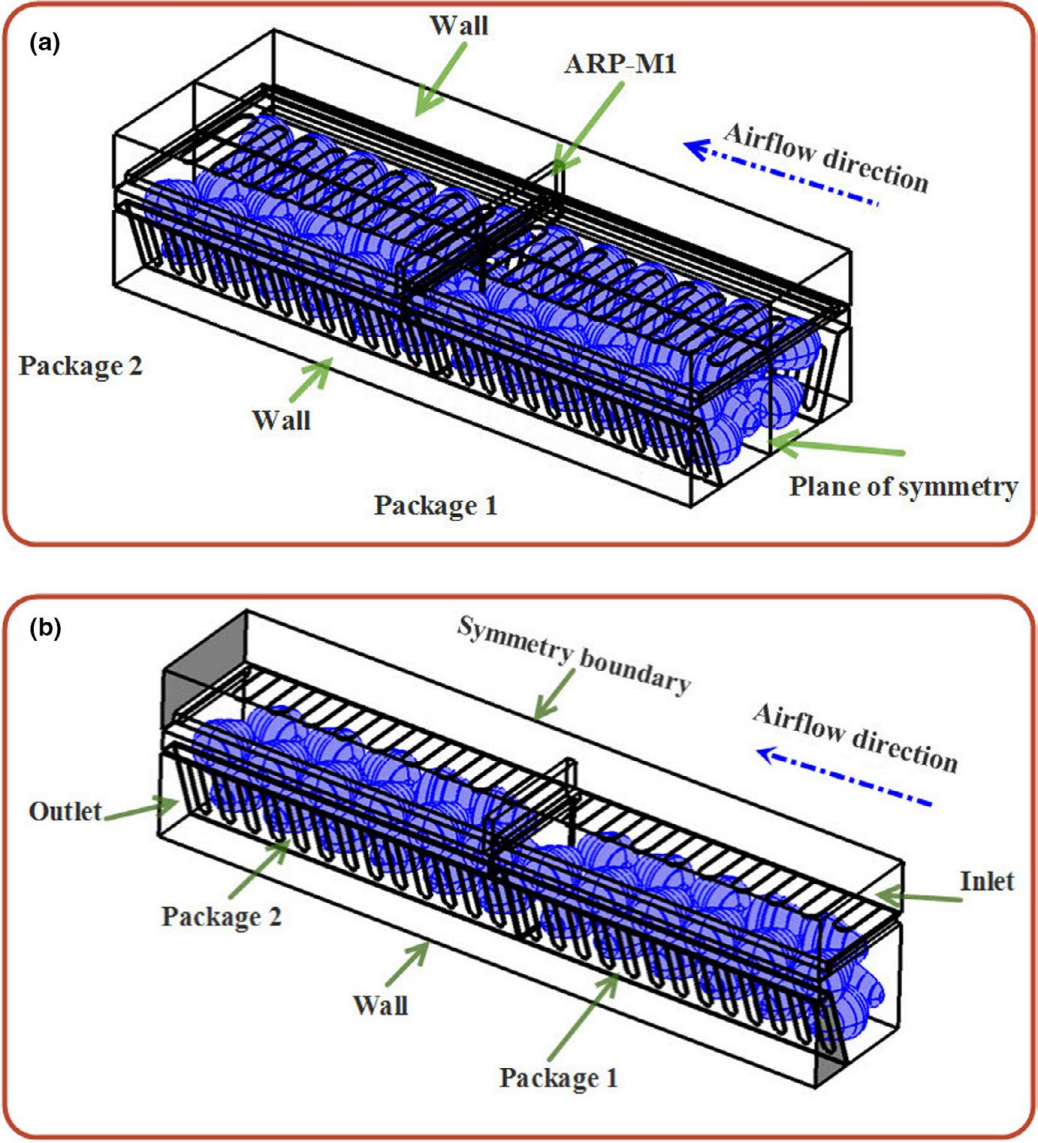
(a) Geometrical model and (b) final computational domain

The computational domain includes two packages placed inside the tray close to each other as shown in Figure 5a. However, a half of this domain was used to simulate the precooling process due to the plane of symmetry created in the computational domain. The final computational domain is shown in Figure 5b. Mesh density was determined according to the mesh independence study. Four meshes were used each containing 96,470, 106,449, 150,890, and 240,532 elements. The results indicated that when the number of elements was above 150,000, the results of numerical solution have acceptable accuracy independent of the number of elements and solution. A finer mesh was used in the critical areas, such as inlet and outlet vents, between the fruit and the fruit–air interfaces. Therefore, the mesh with 160,000 tetrahedral elements (Lagrange quadratic) was adequate for accurate numerical prediction. COMSOL MULTIPHYSICS software (version 3.5) was used to simulate heat transfer and airflow using finite element method and isotropic diffusion. A tuning parameter of 0.5 was selected to prevent numerical instability based on the previous study varied out by (Nalbandi et al., 2016). Simulation was performed using a personal computer with 32 GB RAM.

### 2.3 Extra vents

**FIGURE 6:**
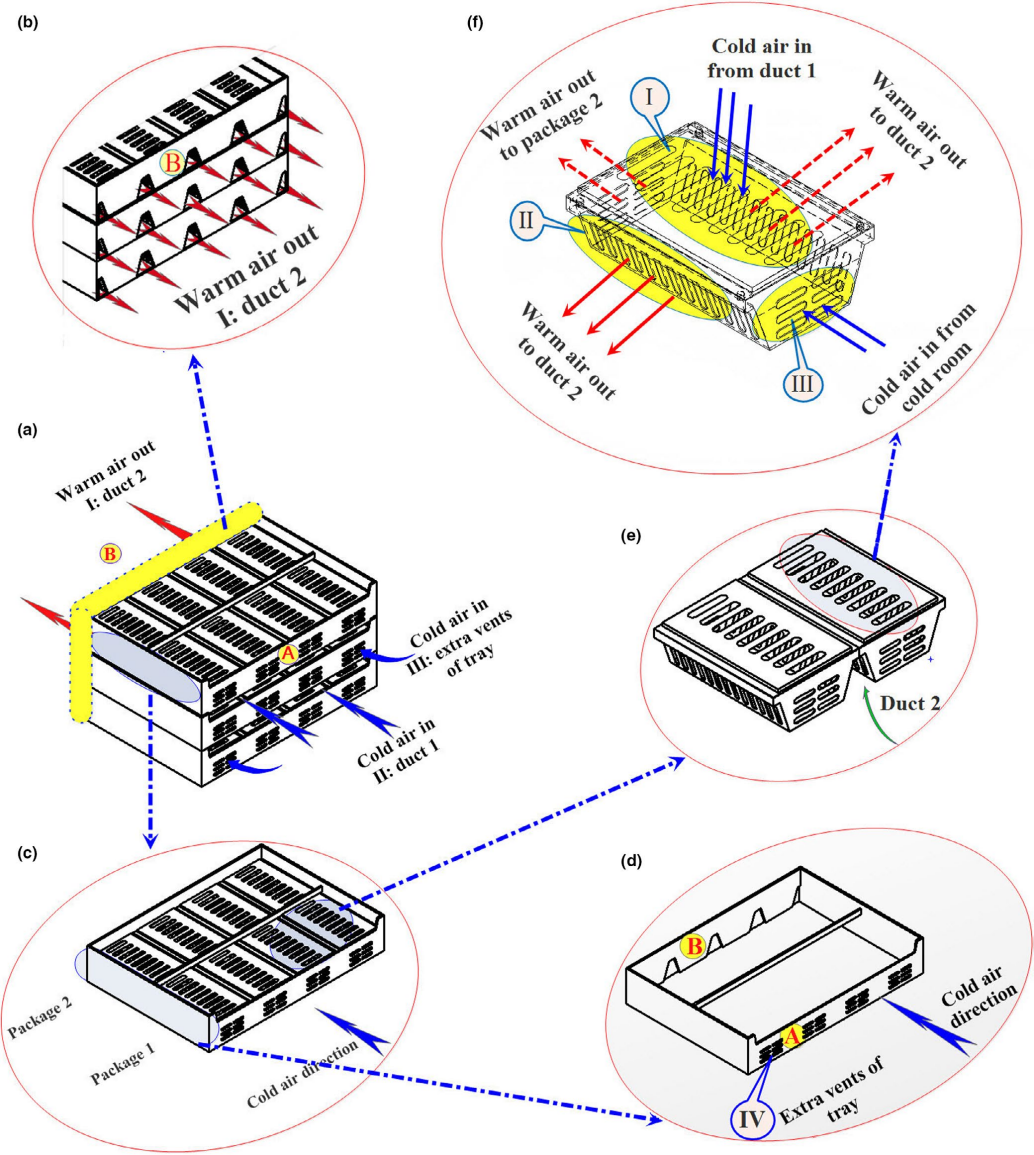
Improved parallel airflow system (IPAS); III in (f) denotes the extra vents of package, and IV in (d) shows the extra vents of tray

It was observed that inserting an airflow restriction plate reduced cooling heterogeneity; however, it failed to deliver enough airflow to the first half of both packages and there was still some heterogeneity in this region (results are discussed in Section 3.1). As shown in Figure 2f, the packages have 26 vents in the lateral walls (each wall consists of 13 vents; Figures 2f‐II) and 9 vents in the package lid (Figure 2f‐I). The total opening area was about 28.2% of the package area. The tray has five vents associated with the opening area of ducts (B in Figure 2d). To increase the cooling uniformity, some extra vents were designed on other sidewalls of the packages (Figures 6f‐III; 6 vents with 0.004 m^2^ area). The total package area increased to 33%. In addition, extra vents were created on side A of the tray wall (Figures 6d‐IV). By these extra vents, the airflow was split into two streams, one of which entered the packages through duct 1 (Figure 6a, I) and the other entered package 1 through the extra vents of tray (Figure 6a, III) from the cold room without passing through duct 1.

**FIGURE 7:**
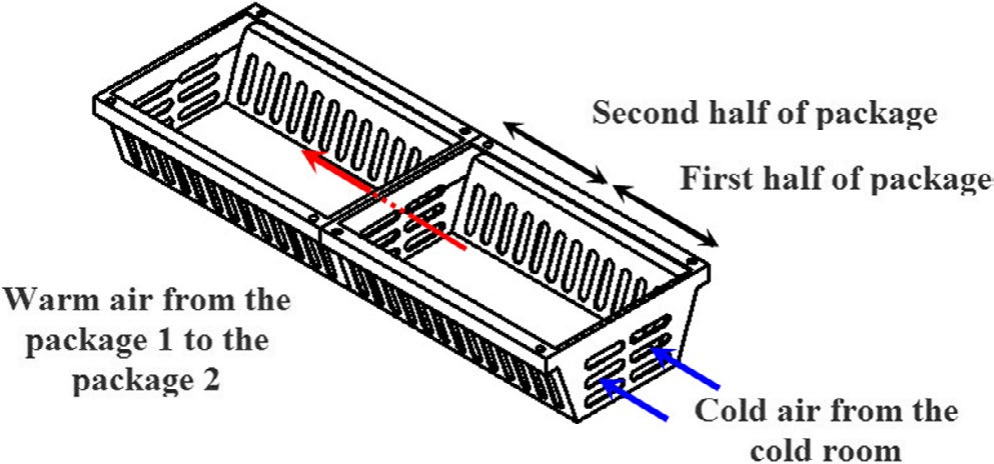
Air path through the extra vents of packages in the improved parallel airflow system (IPAS); the blue arrows show the cold air stream, and the red ones indicate the warm air stream

As shown in Figure 7, a part of the airflow stream in the cold room entered package 1 through the extra vents and simultaneously a part of the exiting airflow from package 1 left it through the vents on the other side and entered package 2. Thus, the first half of both packages received higher amounts of airflow than the previous design, ARP‐M. This new system is introduced as the IPAS, for IPAS, which revised computational domain, and mesh parameters were defined as discussed in Section 2.2. The cooling process of strawberries was simulated based on the revised mathematical models (Table 1).

### 2.4 The effect of moisture loss

The effect of moisture loss was studied by the modified developed models with equations presented in Table 1, and the term of moisture loss was added to the boundary condition of the heat transfer equation (Equation 4 in the product domain (Becker, Misra, & Fricke, 1996; Hoang, Verboven, Baelmans, & Nicolai, 2003; Hu & Sun, 2000). Therefore, the equation of boundary condition was revised as Equation 5, where L is latent heat of evaporation (J/kg) obtained from Equation 6. The term of $\dot{m}$ represents the rate of product moisture loss (kg s^‐1^ m^−2^) that occurs due to the vapor pressure gradient between the fruits and ambient atmosphere, which is calculated by Equation 7. The water vapor pressure at the surface of the product and ambient water vapor pressure are shown by *p*_s_ and *p*_a_ and calculated with Equations 8 and 9, respectively. *P*_w_ is saturation water vapor pressure (Pa) as a function of temperature (Equation 10). Mass transfer coefficient in the product surface (k_g_) is related to the air film mass transfer coefficient ( $k_{g\;\;\text{conv}}$ ) and fruit skin mass transfer coefficient ( $k_{g\;\;\text{skin}}$ ), which are calculated from Equation 11.

The air film mass transfer coefficient is a function of airflow rate and was estimated by using Sherwood–Reynolds–Schmidt correlations (Equations 12, 13 and 14). By calculation of the Sherwood number, Sh (Equation 10), k_m_ was obtained from Equation 15 and finally $k_{g\;\;\text{conv}}$ was calculated by Equation 16. Fruit skin mass transfer coefficient was obtained from lecture (Becker et al., 1996). It describes the skin diffusional resistance to moisture migration.

Finally, the cooling process of strawberries was simulated based on the mathematical modeling of airflow and heat transfer between airstream and the product (Equations 1, 2, 3, and 5) by the COMSOL MULTIPHYSICS software.

(5) $$\left(k_{P}\nabla T_{P}-k_{a}\nabla T_{a}\right)\;\;n=\dot{m}L$$

(6) $$L=9.1T_{p}^{2}-7512.9T_{p}+3875.1\times 10^{3}$$

(7) $$\dot{m}=k_{g}\left(p_{s}-p_{a}\right)$$

(8) $$p_{s}=VPL.\;p_{w}$$

(9) $$p_{a}=RH.p_{w}$$

(10) $$p_{w}\approx \exp\left[23.4795-\frac{3990.5}{T+233.833}\right]$$

(11) $$k_{g}=\frac{1}{\frac{1}{k_{g\;\;\text{conv}}}+\frac{1}{k_{g\;\;\text{skin}}}\;}$$

(12) $$\text{Sh}=2+0.522{\text{Re}}^{0.53}{\text{Sc}}^{0.33}$$

(13) $$\text{Re}=\frac{\rho_{a}.u.d}{\mu_{a}}$$

(14) $$\text{Sc}=\frac{\mu_{a}}{\rho_{a}.\;D}$$

(15) $$k_{m}=\frac{\text{Sh}.D}{d}$$

(16) $$k_{g\;\;conv}=\rho_{\alpha }\frac{M_{H_{2}O}}{M_{\alpha }p_{\mathrm{atm}}}k_{m}$$

### 2.5 Validating the model with the term of moisture loss

**FIGURE 8:**
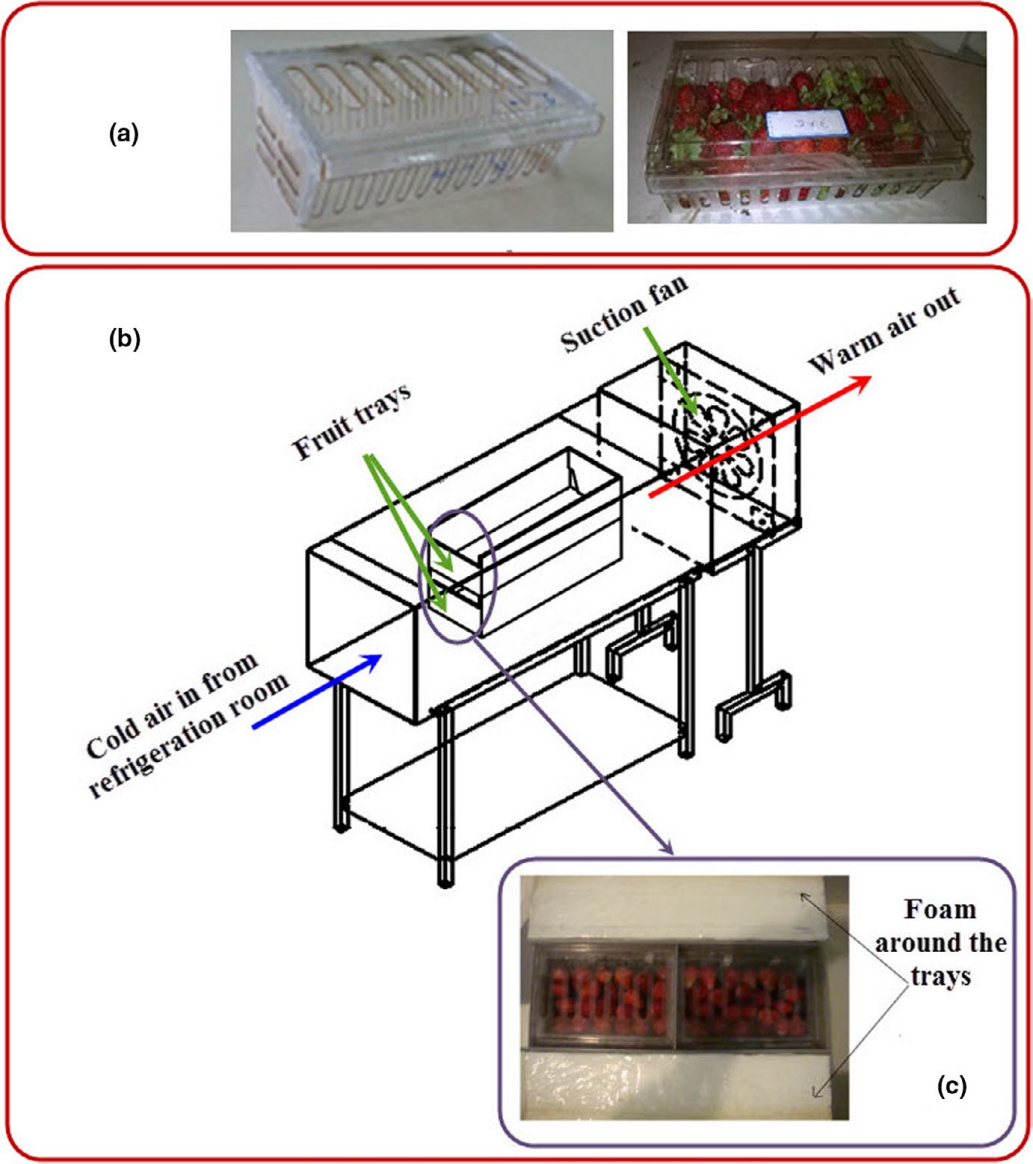
Forced‐air cooling system and fruit trays; (a) fruit package; (b) forced‐air cooling system; (c) isolated tray

**FIGURE 9:**
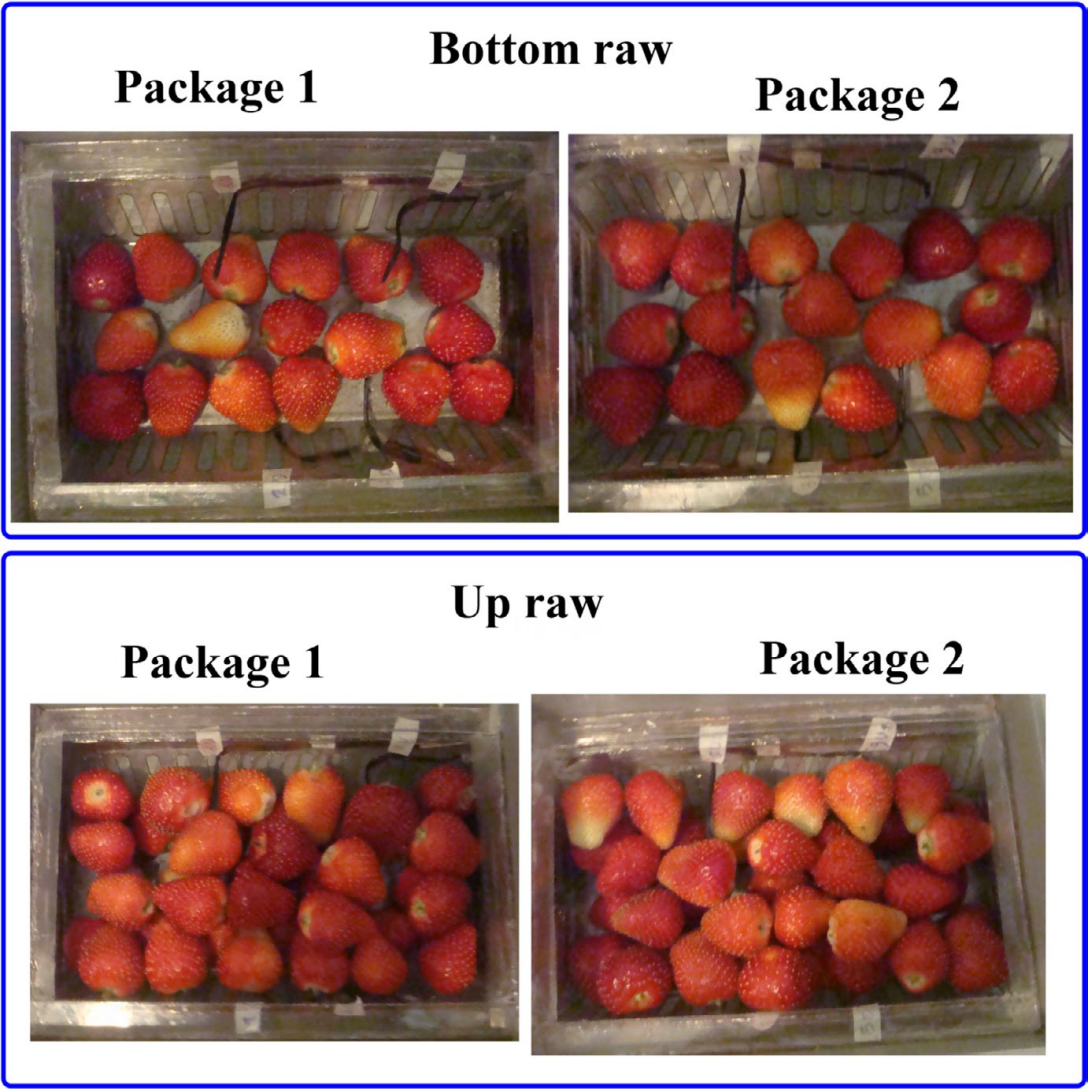
Instrumented strawberries and experimental packages and tray

To validate the model with the term of moisture loss are described in Section 2.4, fresh fruits were purchased from a local greenhouse and cooled immediately using a forced‐air cooling system (Figure 8) placed in a cold room with temperature and relative humidity of 1 °C and 80%, respectively. Strawberries with uniform shape were packed in packages (Figure 8a) according to their arrangement in the simulation process and placed inside the tray. Then, the trays were located in the center of a tunnel (Figure 8b) and their around space was insulated with foam (Figure 8c). Eight strawberries were fitted with K‐thermocouple wire placed in their center (Figure 9). The central temperature of fruit instrumented by thermocouple was compared with those of these positions predicted from the simulator, followed by evaluating the goodness of fit. In addition, the average of central temperature for all instrumented fruits was used as a critical of average temperature of box and compared with that obtained from the simulator. The experiments were conducted at three replications.

## 3 RESULTS AND DISCUSSION

### 3.1 Effect of final design of airflow restriction plate (ARP‐M1)

**FIGURE 10:**
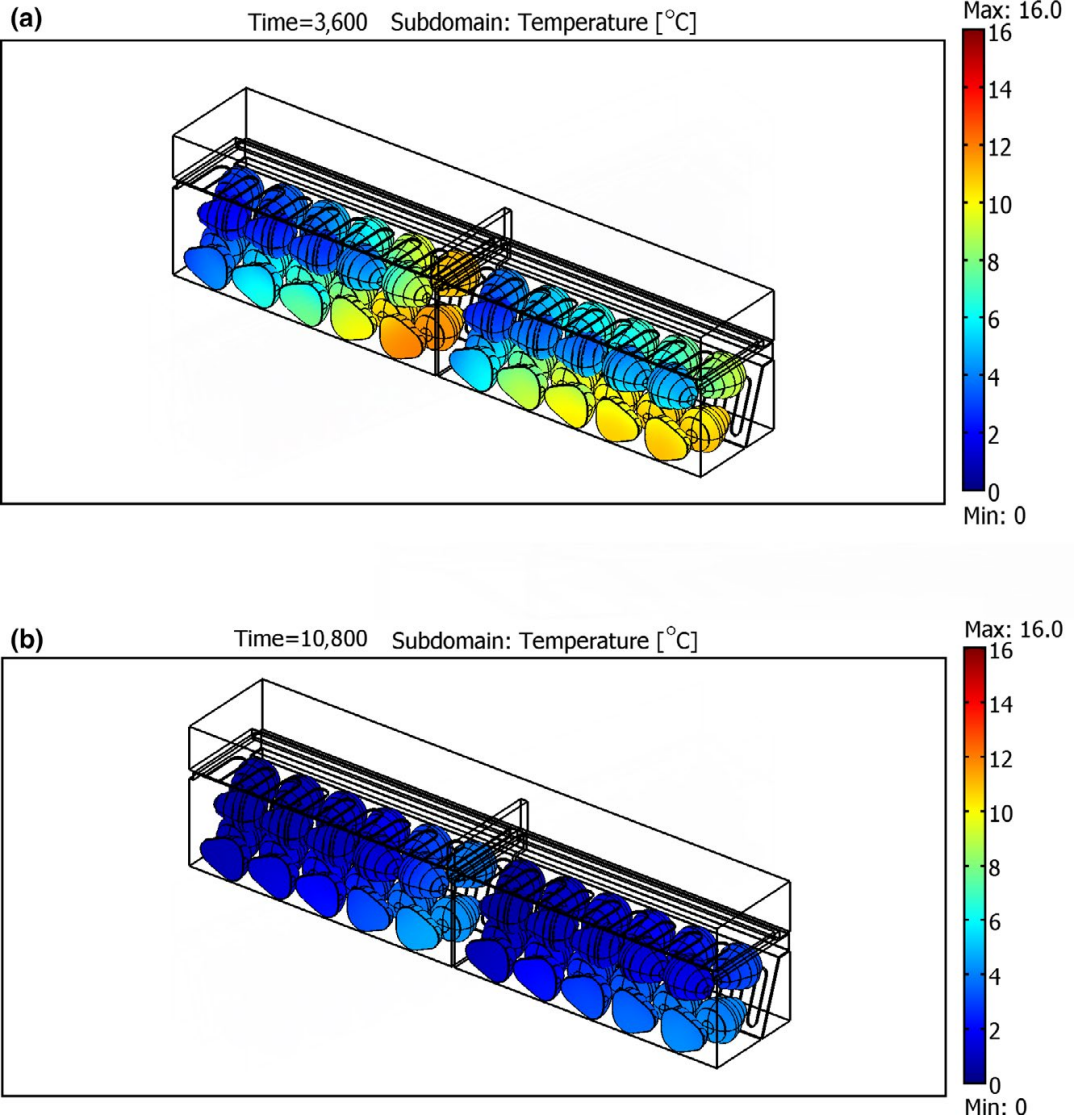
The surface temperature of strawberries within packages 1 and 2 at different times in ARP‐M1; (a) after 3,600 s and (b) after 10,800 s

As clearly shown in Figure 10, final design of airflow restriction plate, ARP‐M1, increased the cooling uniformity among the packages by omitting the dead zone created behind the initial design of ARP. However, it could not maintain the airflow distribution between the packages as the previous design (MPAS; Nalbandi et al., 2016). Table 2 shows the airflow distribution between packages in all the studied systems. In ARP, the first half of packages 1 and 2 received about 43% and 25% of total airflow entered each package, respectively. It was increased in ARP‐M1and reached 42.8% and 37% for packages 1 and 2, respectively, leading to increased cooling uniformity and emitting the dead zone. In addition, the first half of both packages received a low percentage of total airflow and the cooling rate of the fruits located in these zones occurred more slowly. However, it could not maintain the airflow distribution between the packages unlike the MPAS. Therefore, another designing feature was considered to address this problem.

**Table 2:** Cooling time of fruit and airflow distribution in all the designed systems

Kinds of systems	PAS (Nalbandi et al., 2016)		MPAS (ARP) (Nalbandi et al., 2016)		ARP‐M1		IPAS	
Package	1	2	1	2	1	2	1	2
7/8th cooling time (min)	385	235	256	312	316	306	245	245
Total airflow rate (%)	23.6	76.4	48	52	63	37	44.4	55.6
Airflow rate from the first half of inlet vents (%)	50	3.78	43	25	42.8	37	40	33

Abbreviations: ARP‐M1, final design of air restriction plate; IPAS, improved parallel airflow system; MPAS, modified parallel airflow system; PAS, parallel airflow system.

### 3.2 Improved Parallel Airflow System (IAPS)

As discussed in the previous section, modification of the ARP could improve the cooling uniformity; however, some uniformity was observed between fruit temperature. Therefore, some extra vents were designed on the wall of packages and the tray (Figure 6f‐III and 6d‐IV). The results obtained from the simulation process showed that the extra vents provided on the sidewalls of the package and tray had a positive effect on the cooling uniformity, which is discussed in the following section.

#### 3.2.1 Airflow pattern in IAPS

**FIGURE 11:**
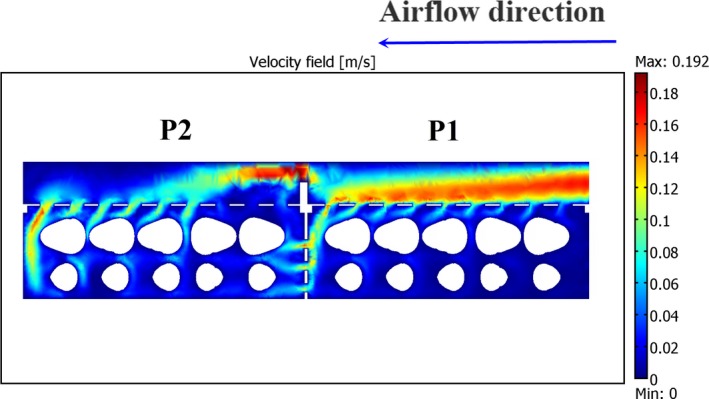
Slice plane plot of the air velocity in improved modified parallel airflow system (extra vents); vertical plane; P1 and P2 represent packages 1 and 2, respectively

The slice plane plot of air velocity is presented in Figure 11. The airflow pattern showed that a part of total airflow entered both packages through the extra vents. In package 1, extra airflow entered from the cold room through the extra vents. In package 2, however, extra airflow was provided from package 1 through the extra vents of both packages. Study of the airflow rate entering both packages indicated that this design could equally split the airflow rate between the packages so that packages 1 and 2 received 44.4 and 55.6% of the total airflow rate, respectively. In addition, the airflow distributions in the first and second halves of package 1 were 40 and 60%, and their corresponding values were 33 and 67% for package 2, respectively. Moreover, about 3.24% and 22% of the total airflow rate entered packages 1 and 2, respectively, through the extra vents. It was clear that the total airflow‐entering package 2 through the extra vents was higher than that for package 1.

#### 3.2.2 Airflow temperature in IAPS

**FIGURE 12:**
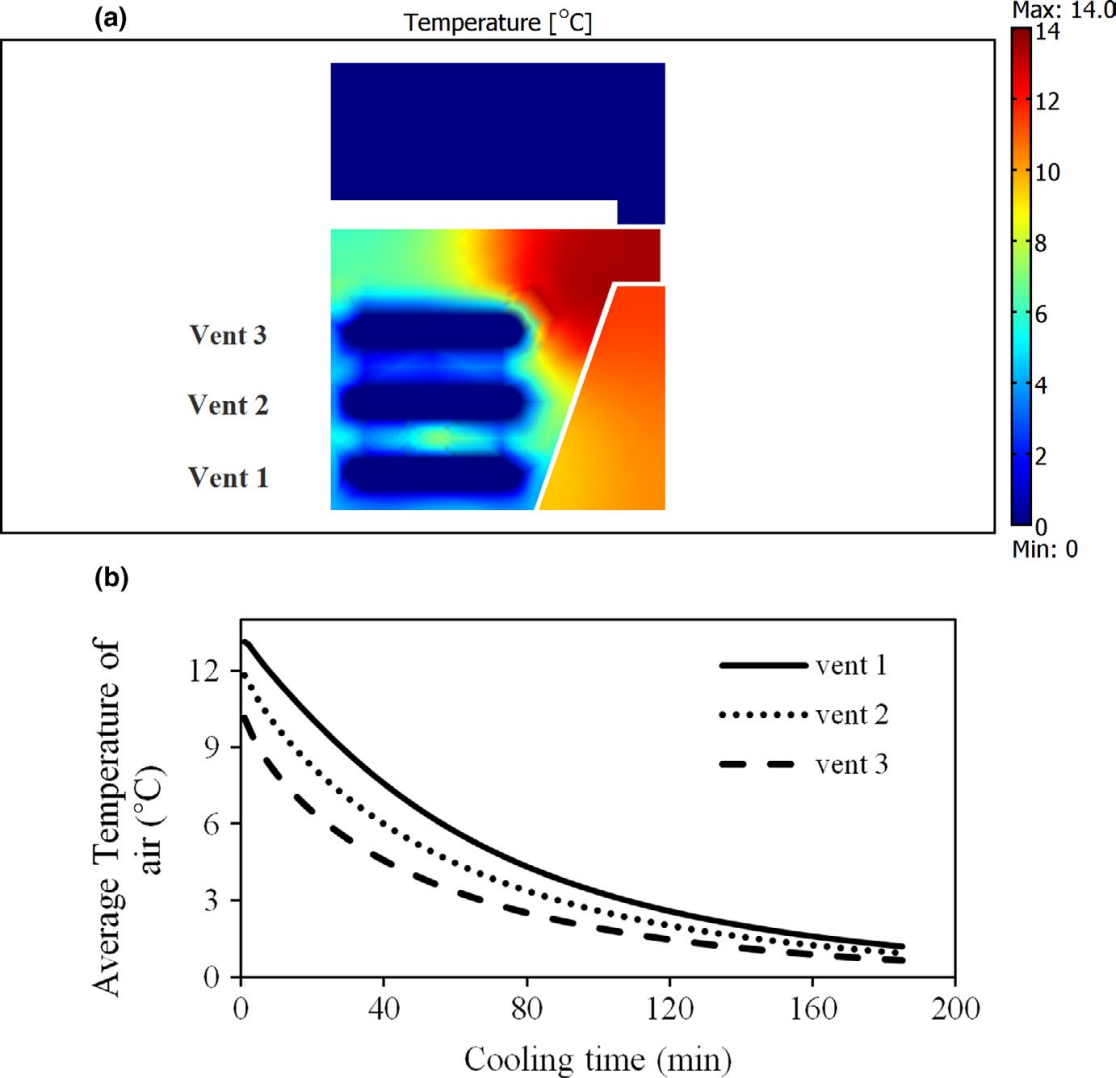
The temperature of air entering the packages through extra vents in improved modified parallel airflow system; (a) package 1, (b) package 2

Similar to the airflow rate, the temperature of air entering the packages was very important. The air temperature entering packages through the extra vents should be studied in terms of the effectiveness of the extra vents in increasing cooling uniformity. The air temperature entering package 1 through the extra vents was always equal to that (1°C) of the cold room (Figure 12a). Nevertheless, it was different for package 2 and related to the convective heat transfer between the airflow and the products inside package 1. Figure 12b shows the air temperature entering package 2 through the extra vents versus time. It is obvious that the air temperature decreased with the progression of cooling due to a low heat transfer inside package 1. Regarding heat transfer, the rate of cooling is a function of air velocity and temperature finally affecting the cooling uniformity.

#### 3.2.3 Average fruit temperature in the packages in IAPS

**FIGURE 13:**
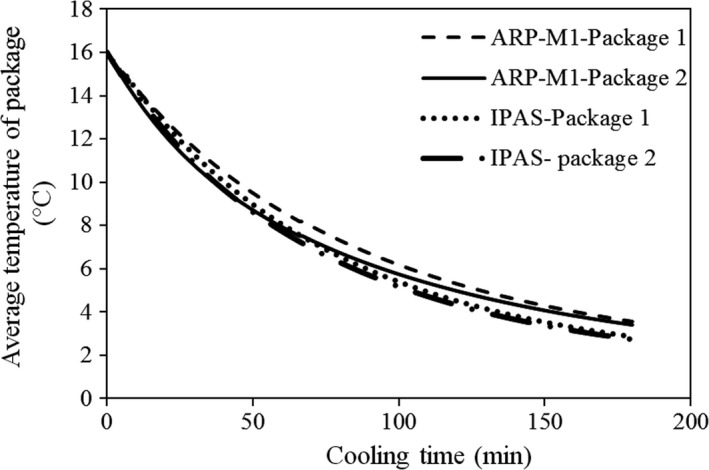
Average fruit temperature of the packages versus cooling time in improved airflow restriction plate (ARP‐M1) and modified parallel airflow system (IPAS)

Average fruit temperature in the packages versus cooling time is shown in Figure 13. As expected from the airflow rate and temperature, the cooling of fruits in both packages was performed with a considerable uniformity. As a result, a temperature difference of 0.1°C was observed between the average fruit temperatures in the packages after 3h of cooling. This difference was constant during the process in spite of the previous design (ARP‐M1) in which it was reducing during the process. Mercier et al. (2019) reported a 7°C difference between the fruit temperature at the end of precooling process using the commercial forced air precooling of strawberries.

**FIGURE 14:**
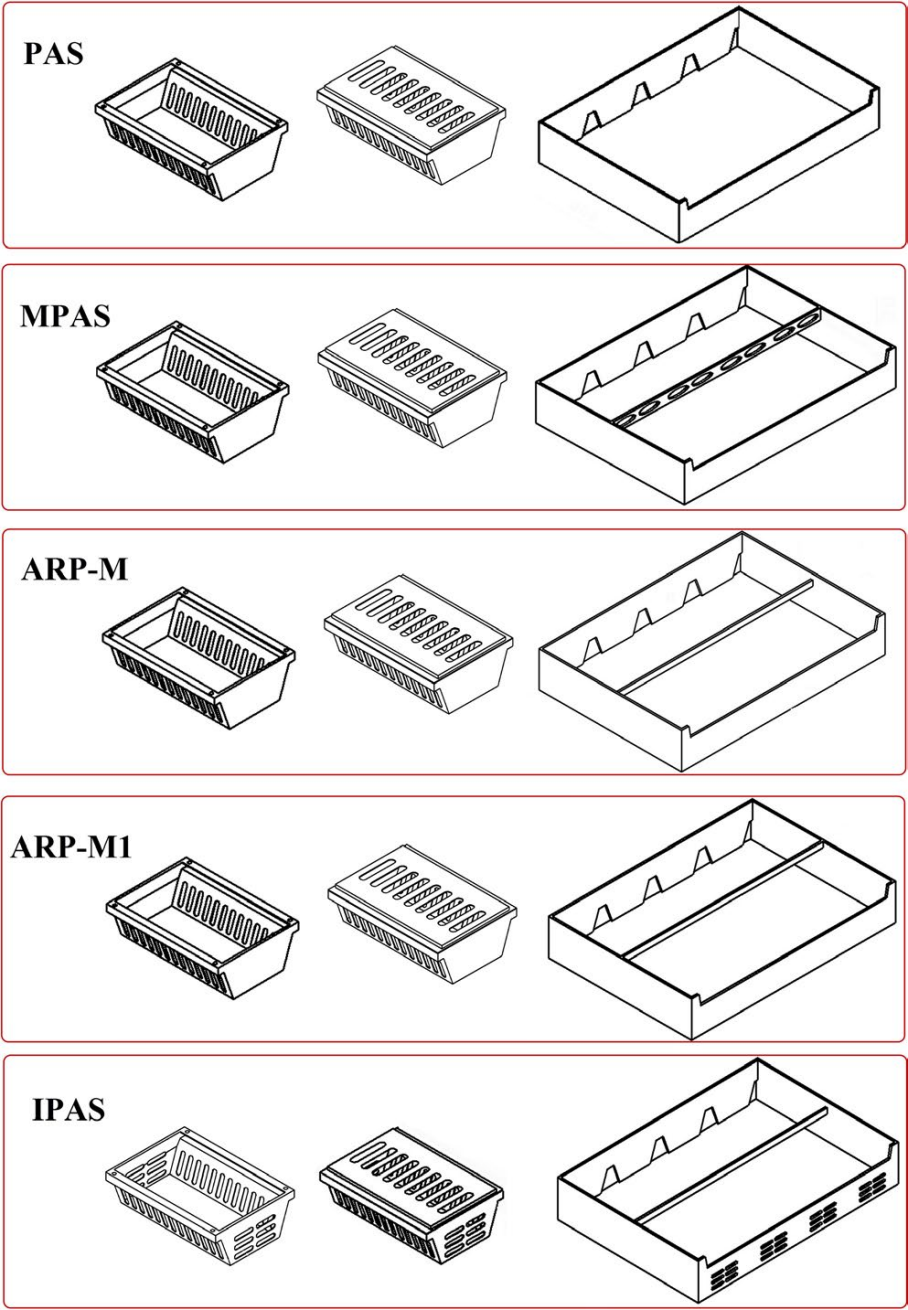
Summary of package and tray designs in the various studied systems; PAS and MPAS are reported from Nalbandi et al. (2016)

The 7/8th cooling time of both packages was about 245 min. Therefore, the cooling time of fruits decreased by using suitable designs. Based on these observations, the cooling time and heterogeneity decreased by using the extra vents. The cooling times of fruits in all designed systems (Figure 14) are presented in Table 2. In industrial systems, there was about 60‐min difference between the average fruit temperature of the packages (Ferrua & Singh, 2009a).

### 3.3 Effect of moisture loss

**FIGURE 15:**
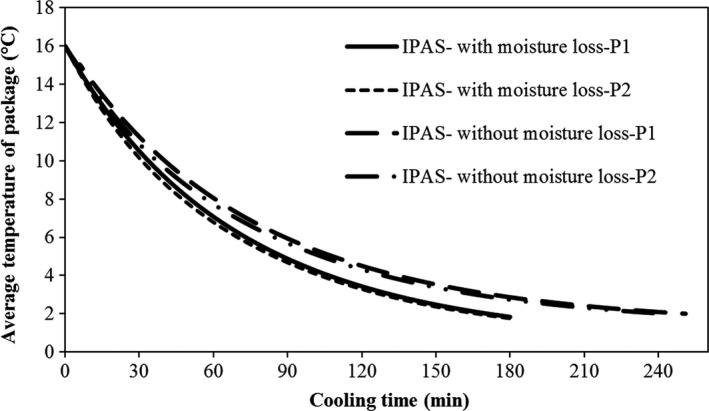
Average temperature of fruits in improved parallel airflow system (IPAS) with and without moisture loss term; P1 and P2 represent packages 1 and 2, respectively

As expected, adding moisture loss term to the mathematical models had a considerable effect on an increase in cooling rate and decrease in cooling time, resulting from the effect of latent heat on cooling of fruit. Based on the results predicted by the simulator, the 7/8th cooling time of fruit (about 165 min) was 75 min lower than that of without moisture loss term (a decrease about 31%). Moisture loss only affected the cooling rate and time but did not affect the cooling uniformity. The cooling uniformity was obtained the same as previously developed model where the moisture loss was assumed negligible. The temperature variation of fruit is shown in Figure 15.

### 3.4 Validation of the developed model with the term of moisture loss

**FIGURE 16:**
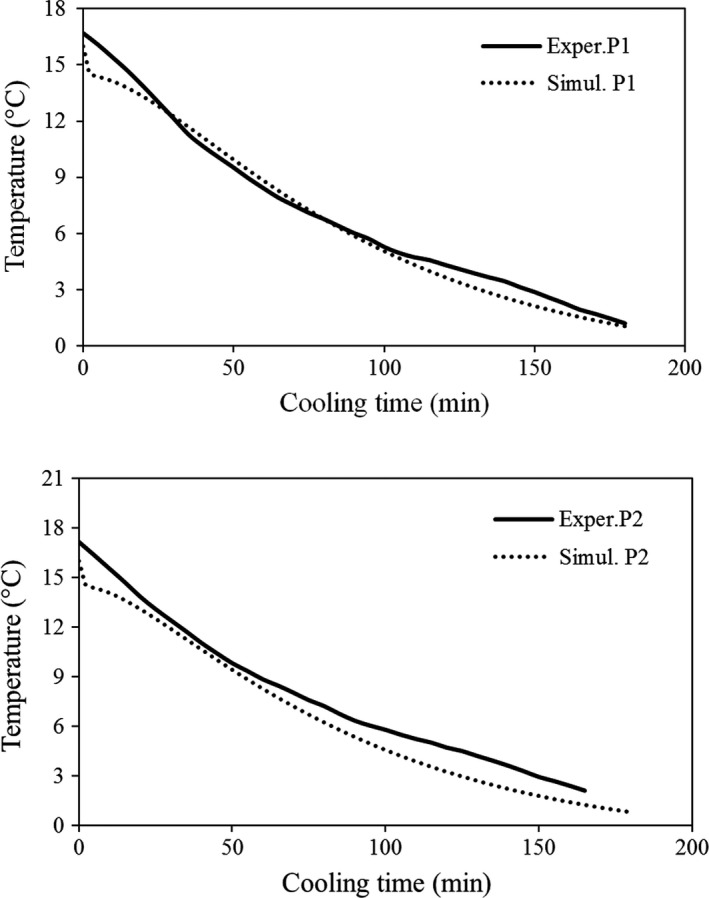
Simulated and experimental central temperatures of instrumented fruits in improved parallel airflow system (IPAS); P1 and P2 represent packages 1 and 2, respectively

In order to validate the new model considering the effect of moisture loss, the fresh fruit was cooled immediately after harvesting. The central temperature of four fruit instrumented with K‐thermocouple wire was compared with that of the same ones obtained from the simulation process (Figure 16). A good agreement was observed between the simulated and experimental data. Therefore, the developed mathematical model with the term of moisture loss and 3D simulator could be used for simulation of precooling process of all fruits, for which the cooling process was performed in the laminar airflow regime.

## 4 CONCLUSION

In the present work, the modified parallel airflow system proposed by Nalbandi et al. (2016) was improved for forced‐air cooling of strawberries. The percentage of opening area increased from 28.2% to 33% by modification of package and tray designs and addition of some extra vents. The new developed system was able to deliver equal airflows to the packages at the same temperature, so that packages 1 and 2 received almost 44.4 and 55.6% of total airflow, respectively. The cooling process of fruits inside both packages was performed more uniformly, and a 0.1°C temperature difference was observed in the average fruit temperature of the packages after 3 hr of cooling process. In addition, the effect of moisture loss as a term was included in the equations to modify the mathematical model. The results showed that moisture loss had a significant effect on the cooling time and rate. Based on the obtained results, this system could be used in the industrial forced‐air cooling of strawberries. This design, however, must be evaluated for two trays, the same as the case for industrial usage.

## NOMENCLATURE

**Table:** 

Cp	Specific heat capacity (J kg^‐1^ °C^‐1^)
D	Diameter of product (m)
D	Diffusivity of water vapor in air (m^2^ s^‐1^)
K	Thermal conductivity (W m^‐1^ °C^‐1^)
kg	Mass transfer coefficient (s/m)
$k_{g_{\mathrm{conv}}}$	Air film mass transfer coefficient (s/m)
$k_{g_{\mathrm{skin}}}$	Skin mass transfer coefficient (s/m)
km	Mass transfer coefficient (m/s)
L	Latent heat of evaporation (J/kg)
Ma	Air molecular mass (kg)
M_H2O_	Water molecular mass (kg)
m	Rate of produce moisture loss (kg s^‐1^ m^‐2^)
n	Outward normal to the surface
P	Pressure (Pa)
pw	Saturation water vapor pressure (Pa)
Re	Reynolds number
Sh	Sherwood
Sc	Schmidt
t	Time (s)
T	Temperature (°C)
VPL	Vapor pressure lowering effect of the product
u	Velocity (m/s)
µ	Dynamic viscosity (Pa.s)
ρ	Density (kg m^‐3^)
Subscripts	
a	Air
atm	Atmospheric
p	Product

## CONFLICT OF INTEREST

Authors declare that they do not have conflict of interest.

## ETHICAL APPROVAL

This study was approved by the University of Tabriz.

## INFORMED CONSENT

Written informed consent was obtained from all study participants.

## References

[fsn31536-bib-0001] Alvarez, G. , & Flick, D. (1999a). Analysis of heterogeneous cooling of agricultural products inside bins. Part I: Aerodynamic Study. Journal of Food Engineering, 39, 227–237.

[fsn31536-bib-0002] Alvarez, G. , & Flick, D. (1999b). Analysis of heterogeneous cooling of agricultural products inside bins. Part II: Thermal study. Journal of Food Engineering, 39, 239–245.

[fsn31536-bib-0003] Amara, S. B. , Laguerre, O. , & Flick, D. (2004). Experimental study of convective heat transfer during cooling with low air velocity in a stack of objects. International Journal of Thermal Sciences, 43, 1213–1221.

[fsn31536-bib-0004] Anderson, B. A. , Sarkar, A. , Thompson, J. F. , & Singh, R. P. (2004). Commercial scale forced air cooling of strawberries. Transactions of ASAE, 47(1), 183–190.

[fsn31536-bib-0005] Becker, B. R. , Misra, A. , & Fricke, B. A. (1996). Bulk refregeration of fruit and vegetables part I: Theoretical considerations of heat and mass transfer. HVAC&R Research, 2, 122–134.

[fsn31536-bib-0006] Brosnan, T. , & Sun, D. (2001). Precooling techniques and applications for horticultural products: A review. International Journal of Refrigeration, 24, 154–170.

[fsn31536-bib-0007] Chakraverty, A. , & Paul, S. R. (2001). Postharvest technology: Cereals, Pulses and Vegetables. India: Sci. Publ.

[fsn31536-bib-0008] Ferrua, M. J. , & Singh, R. P. (2009a). Modeling the forced‐air cooling process of fresh strawberry packages. Part I: Numerical model. International Journal of Refrigeration, 32(2), 335–348.

[fsn31536-bib-0009] Ferrua, M. J. , & Singh, R. P. (2009b). Modeling the forced‐air cooling process of fresh strawberry packages. Part II: Experimental validation of the flow model. International Journal of Refrigeration, 32(2), 349–358.

[fsn31536-bib-0010] Ferrua, M. J. , & Singh, R. P. (2009c). Modeling the forced‐air cooling process of fresh strawberry packages. Part III: Experimental validation of the energy model. International Journal of Refrigeration, 32(2), 359–368.

[fsn31536-bib-0011] Ferrua, M. J. , & Singh, R. P. (2009d). Guidelines for the forced‐air cooling process of strawberries. International Journal of Refrigeration, 32(8), 1932–1943.

[fsn31536-bib-0012] Ferrua, M. J. , & Singh, R. P. (2011). Improved airflow method and packaging system for forced‐air cooling of strawberries. International Journal of Refrigeration, 34, 1162–1173.

[fsn31536-bib-0013] Hoang, M. L. , Verboven, P. , Baelmans, M. , & Nicolai, B. M. (2003). A continuum model for airflow, heat and mass transfer in bulk of chicory roots. Transactions of ASABE, 46, 1603–1611.

[fsn31536-bib-0014] Hu, Z. H. , & Sun, D. W. (2000). CFD simulation of heat and moisture transfer for predicting cooling rate and weight loss of cooked ham during air‐blast chilling process. Journal of Food Engineering, 46, 189–197.

[fsn31536-bib-0015] Kader, A. A. (2002). Postharvest technology of horticultural crops, (3rd ed., p. 3311). Oakland, CA: University of California, Division of Agriculture and Natural Resources Publication.

[fsn31536-bib-0016] Kumar, R. , Kumar, A. , & Murthy, U. N. (2008). Heat transfer during forced air precooling of perishable food products. Biosystems Engineering, 99, 228–233.

[fsn31536-bib-0017] Liu, L. , Ji, M. , Chen, M. , Sun, M. , Fu, X. , Li, L. , … Zhu, C. (2016). The flavor and nutritional characteristic of four strawberry varieties cultured in soilless system. Journal of Food Science and Nutrition, 4(6), 858–868. 27826436 10.1002/fsn3.346 PMC5090650

[fsn31536-bib-0018] Manganaris, G. A. , Iliasb, I. F. , Vasilakakisa, M. , & Mignanic, I. (2007). The effect of hydrocooling on ripening related quality attributes and cell wall physicochemical properties of sweet cherry fruit (Prunus avium L.). International Journal of Refrigeration, 30, 1386–1392.

[fsn31536-bib-0019] Mercier, S. , Brecht, J. K. , & Uysal, I. (2019). Commercial forced‐air precooling of strawberries: A temperature distribution and correlation study. Journal of Food Engineering, 242, 47–54.

[fsn31536-bib-0020] Mukama, M. , Ambaw, A. , & Opara, U. L. (2019). A virtual prototyping approach for redesigning the vent‐holes of pomegranate fruit packaging – a short communication. Journal of Food Engineering, 10.1016/j.jfoodeng.2019.109762

[fsn31536-bib-0021] Nalbandi, H. , Seiiedlou, S. , Ghassemzadeh, H. R. , & Ranjbar, F. (2016). Innovative parallel airflow system for forced‐air cooling of strawberries. Journal of Food and Bioproducts Processing, 100, 440–449.

[fsn31536-bib-0022] O'Sullivan, J. , Ferrua, M. J. , Love, R. , Verboven, P. , Bart Nicolaï, B. , & East, A. (2016a). Modelling the forced‐air cooling mechanisms and performance of polylined horticultural produce. Postharvest Biology and Technology, 120, 23–35.

[fsn31536-bib-0023] O'Sullivan, J. , Ferrua, M. J. , Love, R. , Verboven, P. , Bart Nicolaï, B. , & East, A. (2016b). Forced‐air cooling of polylined horticultural produce: Optimal cooling conditions and package design. Postharvest Biology and Technology, 126, 67–75.

[fsn31536-bib-0024] Wu, W. , Haller, P. , Cronje, P. , & Defraeye, T. (2018). Full‐scale experiments in forced‐air precoolers for citrus fruit: Impact of packaging design and fruit size on cooling rate and heterogeneity. Biosystems Engineering, 169, 115–125.

